# Supplementary figures and images for: TNF signaling mediates cellular immune function and promotes malaria parasite killing in the mosquito Anopheles gambiae
Source: PLoS Pathog. 2025 Jul 3;21(7):e1013329. doi: 10.1371/journal.ppat.1013329 (PMC12244535; doi:10.1371/journal.ppat.1013329)

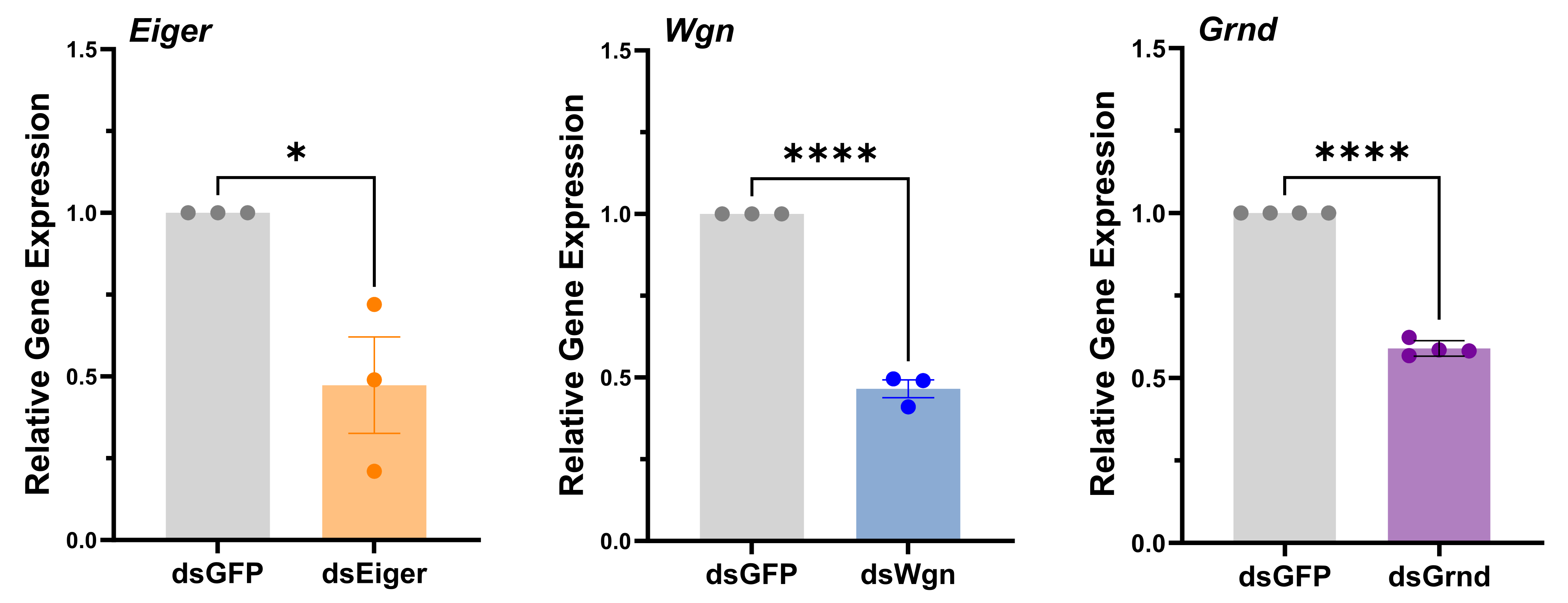

Supplement: S1 Fig — Naïve adult female mosquitoes were injected with dsRNA targeting GFP (control), Eiger, Wgn, or Grnd. Two days post-injection, whole-body mosquitoes (10–15 total) were collected for RNA extraction, followed by gene expression analysis using qPCR. Data from three or more independent experiment were examined for statistical significance using an unpaired student’s t-test. Asterisks indicate significance (* P < 0.05, **** P < 0.0001). (TIF) [file ppat.1013329.s001.tif]

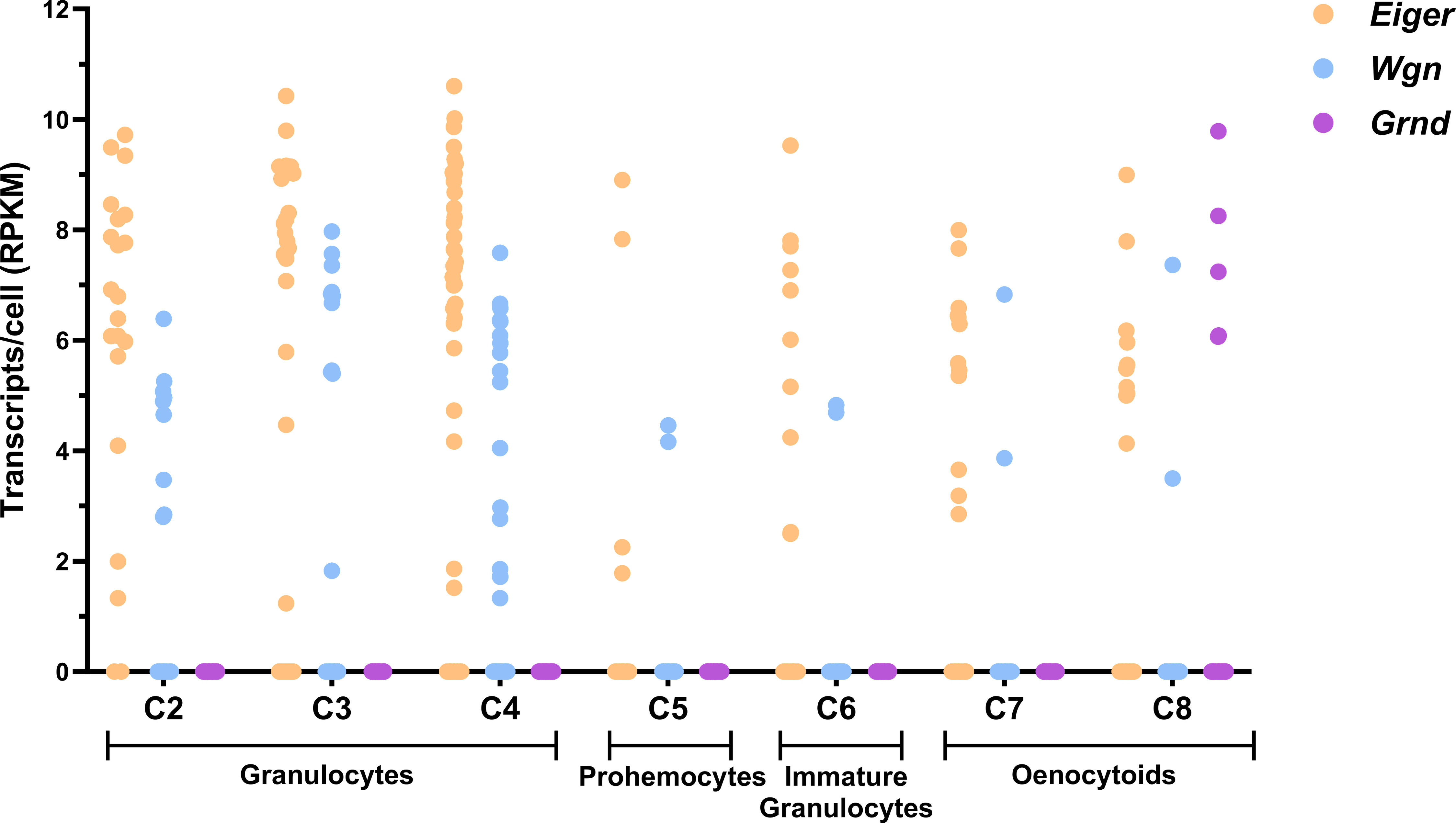

Supplement: S2 Fig — Using previously published single-cell RNA-seq data of An. gambiae hemocytes [22], the expression of Eiger, Wgn, and Grnd was evaluated for each mosquito immune cell subtype. Each dot represents a cell-specific gene expression value. RPKM, Reads Per Kilobase Per Million. (TIF) [file ppat.1013329.s002.tif]

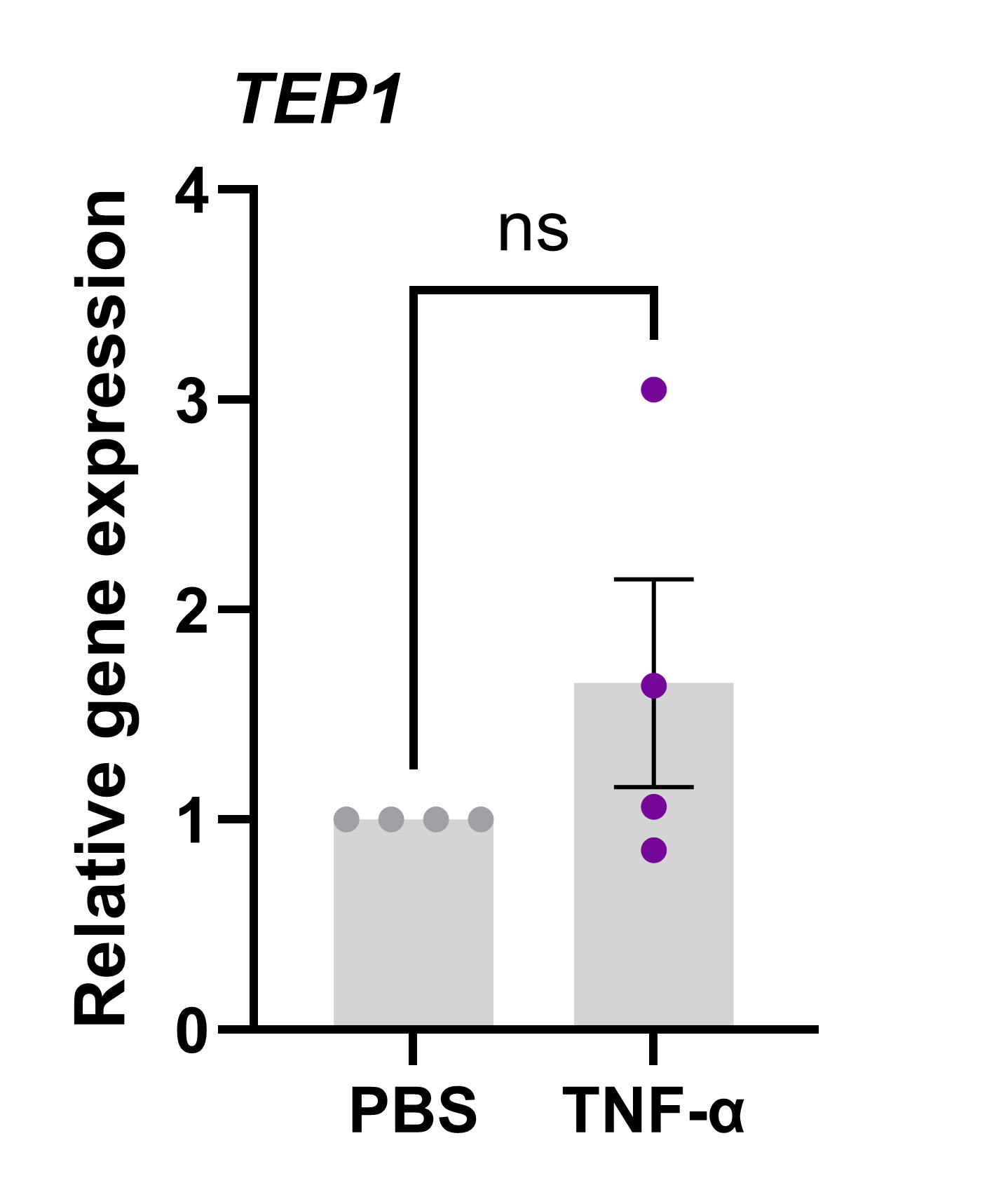

Supplement: S3 Fig — TEP1 expression following the injection of rTNF-α. The influence of TNF signaling on TEP1 expression was assessed in whole mosquitoes, 24 hours after the injection of 1X PBS (control) or rTNF-α injection (50 ng/mosquito). rTNF-α. Data were collected from four independent experiments, each involving 10 female mosquitoes per replicate. Statistical analysis was performed using an unpaired Students’ t-test. ns, not significant. (TIF) [file ppat.1013329.s003.tif]
