# Supplementary material for: TNF signaling mediates cellular immune function and promotes malaria parasite killing in the mosquito Anopheles gambiae
Source: PLoS Pathog. 2025 Jul 3;21(7):e1013329. doi: 10.1371/journal.ppat.1013329 (PMC12244535; doi:10.1371/journal.ppat.1013329)
Supplement: S2 Table — Small letters indicate the T7 promoter sequence. (PDF) [file ppat.1013329.s005.pdf]

S1 Table. List of primers used for gene expression and RNAi. Small letters indicate the T7 promoter sequence.

Gene	Forward (5'-3')	Reverse (5'-3')
Grnd-qPCR	CAAGGCGGTGCCGAAGAATG	GCTTTCCGTCGAATCTTCCG
Grnd-T7	taatacgactcactatagggGTGCGTGTGTGTGTGTTCAG	taatacgactcactatagggCGGACCCTGTTCCTTCTTGT
Eiger-qPCR	TCCGCTGGGATGTAGAAAATCG	GGCGTGGTGGTGCTGTGATG
Eiger-T7	taatacgactcactatagggCAATGAGCTGAACGCTGGAA	taatacgactcactatagggGGCTCGTTGATGGTAAGCTG
Wgn-qPCR	GAGGAGATCCTGTGGGACTG	GCGTCAAAGTGCTTCTCGAT
Wgn-T7	taatacgactcactatagggAAGCATTATCGGCCCAGCTC	taatacgactcactatagggGGGAAGCCAGATTTTGGATCTAG
TEP1-qPCR	CAGACAGATGGTTCGTTTGGTGTG	CCAGCAATGCCGTCAACACATAC
