# Supplementary material for: TNF signaling mediates cellular immune function and promotes malaria parasite killing in the mosquito Anopheles gambiae
Source: PLoS Pathog. 2025 Jul 3;21(7):e1013329. doi: 10.1371/journal.ppat.1013329 (PMC12244535; doi:10.1371/journal.ppat.1013329)
Supplement: Attachment — Submitted filename: TNF-Responses to Reviewers comments.docx [file ppat.1013329.s006.docx]

**Response to Reviewer’s comments**

PPATHOGENS-D-24-00964

"TNF signaling mediates cellular immune function and promotes malaria parasite killing in the mosquito Anopheles gambiae"

A detailed response to each of the reviewer comments is listed below. All changes in response to the reviewer’s comments are highlighted with the “tracking changes” function in the manuscript text.

**Reviewer #1**

***Major Issues***

1. **Eiger appears to the only gene specifically upregulated in Figure 1, and this occurs in Hemocytes and is triggered by both infection and blood feeding. The midgut and carcass is trending towards significance and is likely underpowered at n=6. The authors should increase in the n in this experiment or combine experiments. The authors do not indicate how many times this was done, this needs to be reported.**

Thank you for the comment. We apologize for the lack of experimental detail in our gene expression analysis included in Figure 1. In our experiments, we pooled tissue samples from ~10-30 individual mosquitoes (10-15 for midgut and carcass samples, ~30 for hemocyte perfusions) for each of our experimental samples, such that the data points visualized in our figures represent independent biological experiments (N), not individual mosquitoes (n). We apologize for the confusion. In our revised manuscript, we provide additional experimental details to both the Materials and Methods, as well as the figure legend for Figure 1 and other figures for which we performed similar qRT-PCR analysis.

2. **There is no kinetic looking at whether TNF is playing a role in the initial establishment of infection (i.e. hours after infection), controlling parasite expansion in the mosquito, or whether mosquitoes ultimately clear the infection. The importance of a kinetic is exemplified by the board range of parasite loads observed in individual flies, some flies seem not to impacted by RNAi. Since this is an early description of the pathway an infection kinetic would be warranted.**

While we appreciate the comment, it is unclear what the reviewer means by a “kinetic”, how to address this experimentally, and in what context this relates to our presented data.

Our gene expression analysis of TNF signaling components in Figure 1 was performed by comparing naïve, blood-fed, and *P. berghei*-infected mosquitoes at 24 hours, a relatively standard timepoint by the mosquito community that reflects the physiological changes associated with blood-feeding and the approximate timing of *Plasmodium* ookinete invasion of the mosquito midgut. While the expression of the TNF signaling will undoubtedly change if we examine any developmental stages and experimental timepoints, we believe this information would be extraneous to the general scope of our study examining TNF in parasite infection.

Moreover, our analysis of *P. berghei* infection outcomes relies on the examination of parasite numbers at defined timepoints to determine early- (Day 2) or late-oocyst (Day 8) numbers that have been widely accepted by the mosquito community. With oocyst numbers typically counted by microscopy, this would create an exhaustive amount of work to count oocysts and analyze the knockdowns in gene expression to try to correlate the efficiency of gene-silencing with variable parasite loads in individual mosquitoes.

As a result, it isn’t clear as to how to provide a “kinetic” to further examine TNF signaling in the context of malaria parasite infection in our manuscript in a constructive manner without doing additional experiments that would be outside the scope of this initial study.

With that said, we do agree that future studies should more completely examine the expression of TNF signaling components at different timepoints of mosquito development, tissues, and in the context of bacterial infection.

3. **On line 156 the authors need to demonstrate the efficiency of cell depletion employing this methodology. Rather than stating “which is likely the result” the authors should analyze the impact of CLD treatment on cell numbers.**

Thank you for the suggestion. We have modified the text of our revised manuscript to better state the outcomes of our experiments and have better highlighted the application of clodronate liposomes in our experimental approach. However, we did not repeat these experiments to include assessments of cell numbers following CLD treatment. Yet, in previous studies we have rather exhaustively demonstrated the specificity and function of clodronate liposomes for their application in arthropods (Kwon et al. 2019-*PNAS*; Kwon et al. 2021-*Frontiers in Immunology*; Ramesh Kumar, Smith et al 2021 *Frontiers in Cell and Developmental Biology*; Kwon, Mohammed et al 2021- *eLife*; Adegoke et al. 2023- *Frontiers in Immunology*; Mameli, Samantsidis et al. 2024- *bioRxiv*). This includes manual cell counts, qRT-PCR, flow cytometry, immunofluorescence assays, and genome-wide CRISPR screen to evaluate its function. As a result, we do not believe that is essential to again validate the application of phagocyte depletion using clodronate liposomes.

4. **Perhaps most surprising, the paper lacks any sort of assessment of the impact of the TNF-a pathway on mosquito survival or transmission potential. The authors should assess these parameters to warrant publication. Infection dynamics in infected insects are known determinants of subsequent transmission and disease severity (for example see Courtney et. al. PLoS Path 2017). The pathway appears to be active, impacts parasite numbers, but is it actually protective, increasing mosquito survival and does it have downstream impacts of transmission and subsequent disease.**

Thank you for the suggestion. In our revised manuscript, we added new survival experiments that examine the survival of Eiger, Wengen, and Grindelwald knockdown (RNAi) phenotypes under naïve conditions performed at both 19 and 27°C. In addition, we also examine survival following P. berghei infection. These data have now been included in a revised Fig 2. From these experiments, our data suggest that the RNAi-silencing of pathway components does not have significant effects on mosquito survival under naïve conditions, and only Grindelwald displayed a slight fitness effect following *P. berghei* infection.

Regarding the comment on transmission potential, we have tried to search for the suggested paper by Courtney et. al. PLoS Path 2017, however, we have been unable to identify a manuscript by Courtney et al that matches this description. We have also tried to find the relevant paper using key words as provided by the reviewer but to no avail. Without a better understanding of the type of experiments desired by the reviewer to address transmission, we opted to not attempt further experiments.

***Minor Issues***

1. **In the abstract the authors state that the TNF pathway has yet to be explored in mosquito innate immunity. While the paper provides new information and there is more to learn about immunity in vector insects, this is an overstatement and the authors should give better acknowledgement to:**
   [**https://www.nature.com/articles/s41598-022-23780-y**](https://www.nature.com/articles/s41598-022-23780-y) **(Particularly on lines 95-97).**

We apologize for the oversight. In our revised manuscript, we now mention this study in the introduction to better illustrate the previous identification of TNF signaling components in *Anopheles* mosquitoes (Lines 100-103).

2. **The information provided in the Mat and Meth regarding the mosquito infections need more detail.**

Additional experimental details have been provided in the Materials and Methods of our revised manuscript (Lines 460-469).

3. **Figure 2, B and C are using the same control group and should be graphed as such.**

This has been modified as suggested in our revised manuscript.

4. **On line 156 the authors begin to describe experiments employing CLD, The authors need to define what the intent of the use of CLD.**

We have modified this section of text for clarity to better define our intent and reasoning for using CLD to resolve the expression of *Wgn* and *Grnd* in hemocyte subtypes in our revised manuscript (Lines 200-208).

5. **The logical jump from the use of previous transcriptomic data to Figure 4A on line 158 is not tenable. There has to be some representation of the actual data that leads to this conclusion.**

We have modified this section of text in our revised manuscript to enhance clarity and to better define how we used previous single-cell data to develop a working model for the expression of *Wgn* and *Grnd* in hemocyte subtypes (Lines 194-208).

6. **The authors state “Given the absence of Grnd in granulocytes” This is gene expression, the authors need to actually show the receptor is not present.**

Thank you for the suggestion. Unfortunately, in mosquitoes we are severely limited by the lack of antibodies or other markers that would enable methods to demonstrate the presence/absence of Wgn or Grnd in mosquito hemocyte subpopulations. As a result, we are forced to heavily rely on gene expression and methods of granulocyte depletion in our study. We now highlight these limitations in our revised manuscript (Lines 194-208).

We do hope to someday generate antibodies in the future to aid in the further dissection of the TNF signaling pathway in mosquitoes, yet at present this is a significant investment of time and resources that would further delay publication and would extend beyond the scope of our manuscript.

7. **The data in 4F requires better explanation, the authors that that the gene expression is associated with oenocytoids and that rTNF-a expression loss indicates lysis, but it could also indicate down-regulation, the authors need to simply quantify the oenocytoids, they are relying too heavily on gene expression. An assessment of oenocytoid apoptosis, cell death may shed further light on the impact of rTNF-a.**

As suggested, we have added new data in which we quantify the percentage of oenocytoids (based on morphology using a hemocytometer) following rTNF-a injection in our revised manuscript (now Figure 4F). While we consider this type of quantification somewhat arbitrary due to the absence of reliable markers to accurate distinguish immune cell subtypes, these data suggest that the injection of rTNF-a significantly reduces the percentage of oenocytoids, providing further support that rTNF-a promotes oenocytoid lysis.

At present, a further assessment of oenocytoid lysis is very challenging. Oenocytoids are believed to rupture in ~40 minutes or less following external stimuli, and this cell type doesn’t readily adhere to glass slides to attempt more detailed microscopy. At present, without a oenocytoid specific marker, the suggested experiments to examine apoptosis of this cell type in mosquitoes are near impossible.

8. **The authors need to ensure they are doing a post-test correction for comparisons between more than 2 groups.**

While it is unclear if the reviewer comment is specific to one or more data sets presented in our manuscript, all statistical analysis involving three or more data sets incorporates a multi-test correction. For instance, our oocyst infection data was analyzed using Kruskal-Wallis and Dunn’s post-test to determine significance. In our revised manuscript, we have taken additional steps to ensure that more detailed descriptions of our statistical analysis are included in the figure legends.

**Reviewer #2**

***Major Issues***

1. **The authors used an ectopic human TNF molecule, the r-α TNF to, theoretically, promote mosquito TNF signaling. However, they bring no evidence that the r-α TNF effect is mediated by the mosquito TNF signaling pathway (i.e. via Wgn and Grnd)? For example, is this r-α TNF effect abolished in dsWgn or dsGrnd backgrounds? Otherwise, what is the relevancy of using an ectopic human TNF molecule, if we are not sure it activates the mosquito TNF pathway? This is important because the same r-α TNF is also used to address the influence of mosquito TNF pathway on the granulocytes and the phenoloxidase activity later in the manuscript.**

Thank you for the suggestion. We have performed additional RNAi experiments to demonstrate that the anti-*Plasmodium* effects observed following the injection of rTNF-a are indeed mediated by Wengen and Grindelwald. These data are now included in a revised Figure 4A.

2. **The authors mentioned that at least 3 independent replicates were performed for most figures. However, the n number of mosquitoes, representing the total number of the 3 replications, looks very weak. For example, in Figure 2A n=35; Fig.3, dsWgn, n=26. In the latter case, it would mean that an average of less than 10 mosquitoes/replication was used. 60 mosquitoes for the 3 replications would be the minimal number of mosquitoes that would lead to reliable and robust conclusions.**

We have added additional experimental replicates in our revised manuscript to increase the number of individuals examined in our infection experiments.

3. **The dsWgn condition always presents a weaker number of mosquitoes compared to the dsGFP condition. Does this mean that dsWgn led to a higher mortality rate compared to dsGFP? If this is the case, Wgn would be a vital gene.**

Thank you for the suggestion. In our revised manuscript, we added new survival experiments that examine the survival of Eiger, Wengen, and Grindelwald knockdown (RNAi) phenotypes under naïve conditions performed at both 19 and 27°C, as well as examine survival following *P. berghei* infection. These data have now been included in Fig 2. From these experiments, our data suggest that the RNAi-silencing of pathway components does not have significant effects on mosquito survival under naïve conditions, while only Grindelwald displays a small effect following Plasmodium infection.

4. **Figure 4 aims to illustrate the influence of the TNF pathway on mosquito hemocytes.
   Figure 4D shows that ectopic addition of r-alpha TNF increases the rate of granulocytes. In Fig. 4E, the way statistical analysis was made does not properly highlight the effect of the ectopic r-alphaTNF on granulocytes. For each dsRNA condition, the authors just compared the influence of the ectopic addition of r-alpha TNF on granulocyte rate. It would be more accurate and informative to compare the dsGFP condition (with or without r-alpha TNF) with the corresponding dsWgn and dsGrnd conditions. For example, dsGFP (r-α TNF+) with dsWgn (r-alpha TNF+).
   Another information could be extracted from Figure 4E: although the addition of r-α TNF increased the granulocyte rate, the TNF signaling pathway does not control the basal level of granulocytes: comparing dsGFP with dsWgn in r-α TNF(-) background or comparing dsGFP with dsGrnd in r-α TNF- background. The authors do not mention or at least discuss this.**

Thank you for the comment. We feel that the comparisons displayed in Figure 5E (previously Figure 4E) provide the best representation of our data demonstrating the effect of rTNF-a on granulocyte populations in each genetic background.

When we compare basal levels of granulocytes in each of the RNAi backgrounds following the PBS treatment (Kruskal-Wallis with Dunn’s post-test), we do not see any differences in the percentage of granulocytes (below, left panel). However, we consider this result “inconclusive” as to whether TNF signaling influences basal levels of hemocyte subtypes. For mosquitoes, RNAi remains one of the primary tools in our ability to assess gene function, yet since RNAi was performed in adult mosquitoes, it may not account for potential roles of TNF signaling that occur earlier in mosquito development and hematopoiesis (which remains a relative mystery). We have incorporated additional text in the discussion of our revised manuscript to highlight this limitation.

Similarly, when we examine the effects of rTNF-a treatment on granulocytes from each genetic background, the dsGFP background (as expected), is the only one that is significantly different (on left, right panel). As a result, we do not see the need to alter our current display of the data in Figure 5E.

5. **Figure 4H: if r-alpha TNF passes through Wgn and/or Grnd, why r-alpha TNF+ background is required to get an effect on PPO1 and PPO8? I would also expect to see at least a similar tendency in the PBS background, but the authors do not explain why. It looks like there is no basal activity of the TNF pathway near on the granulocytes nor on the PPO activity, which is unlikely to be in a biological system. How could the authors explain that?**

Thank you for the comment. For the data presented in Figure 5I (previously Figure 4H), we use a heat map to summarize changes in oenocytoid PPO1 and PPO8 gene expression in control (injection of 1x PBS) and experimental conditions (injection of rTNF-a). In these experiments, we see reduced expression of PPO1 and PPO8 in dsGFP controls following rTNFa injection (similar to data presented in Fig 5G), which implicates that the injection of rTNFa has initiated oenocytoid lysis (supported by data in Fig 4F-H). However, when similar experiments are performed in either the dsWgn or dsGrnd backgrounds, this effect is abrogated, suggesting that loss of either Wgn or Grnd is able to prevent oenocytoid lysis.

As a result, we believe that these two receptors may be functioning as a heterodimer to initiate these signals, where the loss of either component disrupts signaling. While several experiments are required to validate this hypothesis, which are beyond the scope of this paper, this would best explain our experimental observations. We have added additional text in our revised manuscript to better reflect this model of TNF signaling on cellular immune function.

6. **Figure 5: Granulocyte depletion via CLD treatment does not completely abolish the effect mediated by r-alpha TNF(+). The authors mentioned the potential implication of humoral response but without testing the hypothesis, by at least looking at the expression level of the complement-like system mediated by APL1C/LRIM1 and TEP1. As a major humoral anti P. berghei immune complex, this would show a linkage between the TNF and the complement-like system in Anopheles mosquitoes.
   In addition, could the author rule out the implication of an additional population of hemocytes than granulocytes that could be involved in the midgut attachment, illustrated in Figure 5B?**

Thank you for the suggestions. In our revision, we have performed additional experiments to examine TEP1 expression following rTNF-a injection and have now included these results as Figure S3. While we do see an increase in TEP1 expression, these results are not significant, and are therefore inconclusive as to whether the increased killing responses are mediated by TEP1 and mosquito complement. While we are very interested in performing additional experiments to identify the molecular mechanisms behind TNF-mediated killing, this will likely require substantial work, and believe that these questions are beyond the scope of this study and are better addressed at a later time.

In addition, regarding the comment of ruling out that another hemocyte population could be responsible for midgut attachment, this is at present a challenging question to address. Granulocytes are most often implicated in midgut attachment, yet with several granulocyte subtypes, it is unclear if this is specific to a specific type of cell or a shared property of granulocytes. Recent studies have also implicated megacytes in midgut attachment, further clouding this type of analysis. Unfortunately, at present, we simply lack the knowledge, tools, or genetic resources to accurately address this question.

7. **Finally, although the author claims in the title and the abstract use a general term such as “Plasmodium” or “malaria parasites”, it is important to note that the experiments were only performed with the rodent malaria parasite, P. berghei but not with the human malaria parasite. Therefore, cautiousness should be considered while using general mentions such as “Plasmodium” or malaria parasite in the text, especially considering the differences in mosquito immune responses described in the literature between rodents versus human malaria parasites. This is mentioned anywhere in the text, suggesting that the authors may consider these findings as a general mechanism for all Plasmodium.spp.**

Our use of “Plasmodium” and “malaria parasites” in general terms in the manuscript is not meant to deceive or hide that our experiments were performed using the rodent malaria model, *Plasmodium berghei*, and tried to make this distinction clear throughout the manuscript. However, we have modified the text in several locations our revised manuscript to remove these generalities to improve clarity and transparency.

In addition, we have also added a paragraph to the discussion of our revised manuscript that reiterate that these studies were only performed using the rodent malaria model and that further work is needed to examine the potential effects on human malaria parasites.

***Minor Issues***

1. **Figure 4 title is not appropriate. I would rather propose: “The TNF signaling pathway influences the granulocyte abundance and prophenoloxidase activities.”**

Thank you for the suggestion. This has been changed as requested in our revised manuscript.

2. **L 250-251: I found interesting the role of TNF-alpha in Drosophila intestinal stem cells and gut homeostasis. Gut homeostasis is also linked with the enteric microbiota, which, in Anopheles mosquitoes, is known as a determinant for Plasmodium infection. It would also be interesting to tackle the potential role of the mosquito TNF signaling pathway in the microbiome.**

Thank you for the comment. While direct roles or interactions with the microbiome on TNF signaling have not yet been identified, we have added additional text to the discussion of our revised manuscript to address this possibility.

3. **Line 300: Replace “demonstrate” with “suggest”, as correctly mentioned in Line 309. The data support this idea but do not demonstrate it.**

This has been corrected in our revised manuscript.

4. **It would be interesting to tackle the influence of a potential TNF-mediating immune priming through a blood meal on a vertebrate host carrying high levels of TNF-alpha: this physiological background is probably well described in the literature where high levels are found in patients with chronic inflammation (polyarthritis), chronic infectious diseases, pregnant women, etc…**

We would like to thank the reviewer for the interesting suggestion. With several previously studies describing the roles of blood-meal derived components acting on mosquito immune function, it is a very interesting possibility that increased levels of mammalian TNF in a human host could have similar effects on mosquito vector competence. We have added a new paragraph to the discussion in which we discuss these possibilities.
